# Supplementary material for: A Novel Method to Prepare Transparent, Flexible and Thermally Conductive Polyethylene/Boron Nitride Films
Source: Nanomaterials (Basel). 2021 Dec 30;12(1):111. doi: 10.3390/nano12010111 (PMC8746404; doi:10.3390/nano12010111)
Supplement: Supplementary file 1 [file nanomaterials-12-00111-s001.zip › nanomaterials-1510032-supplementary.pdf]

nanomaterials

Supporting Information

A Novel Method to Prepare Transparent, Flexible and Thermally Conductive Polyethylene/Boron Nitride Films

Mingming Yi [1,2,†], Meng Han [2,†], Junlin Chen [1], Zhifeng Hao [1,*], Yuanzhou Chen [1], Yimin Yao [2] and Rong Sun [2,3]

[1] School of Chemical Engineering and Light Industry, Guangdong University of Technology, Guangzhou 510006, China; 203701063@csu.edu.cn (M.Y.); 2112106060@mail2.gdut.edu.cn (J.C.); m15521162019@163.com (Y.C.)
[2] Shenzhen Institute of Advanced Electronic Materials, Shenzhen Institute of Advanced Technology, Chinese Academy of Sciences, Shenzhen 518055, China; meng.han@siat.ac.cn (M.H.); ym.yao@siat.ac.cn (Y.Y.); rong.sun@siat.ac.cn (R.S.)
[3] Shenzhen College of Advanced Technology, University of Chinese Academy of Sciences, Shenzhen 518055, China
* Correspondence: haozf@gdut.edu.cn
† These authors contributed equally to this work.

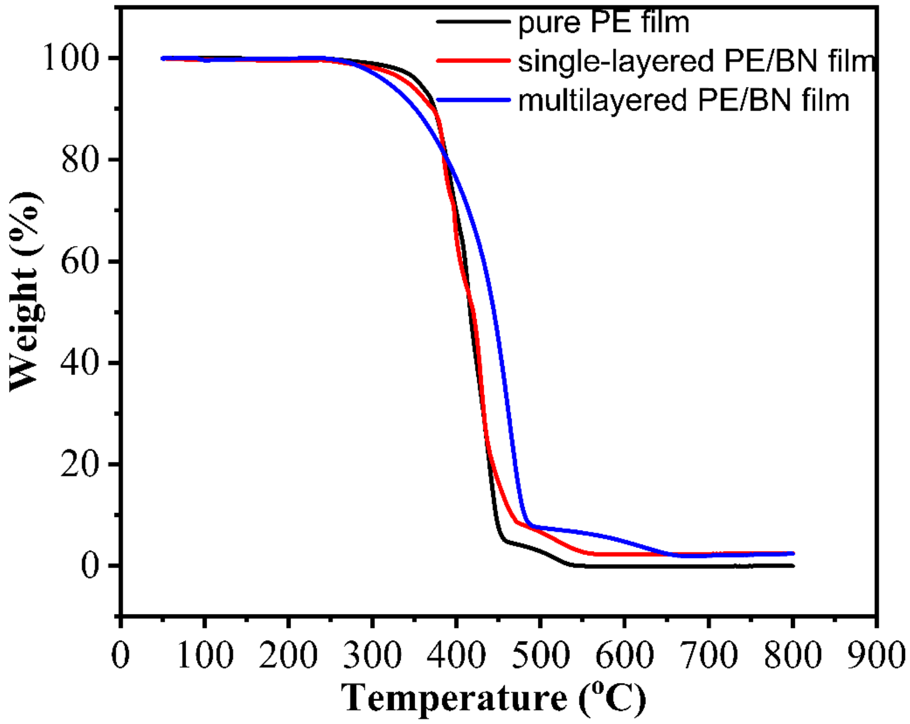

Figure S1. The TGA curve of pure PE film, single-layered PE/BNNPs film and multilayered PE/BNNPs film.

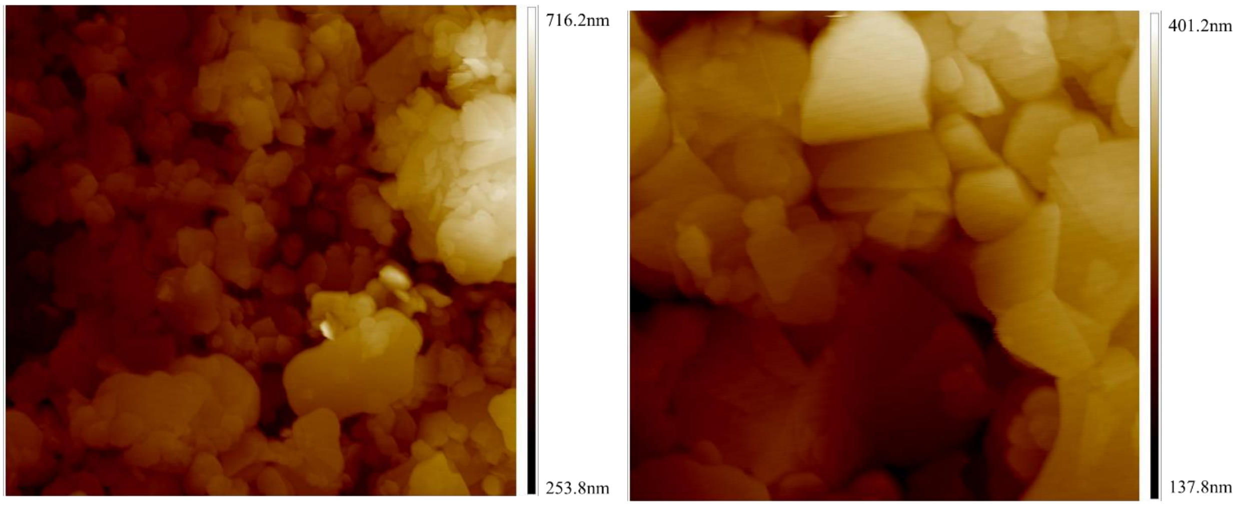

Figure S2. AFM image of single-layered PE/BNNPs film.

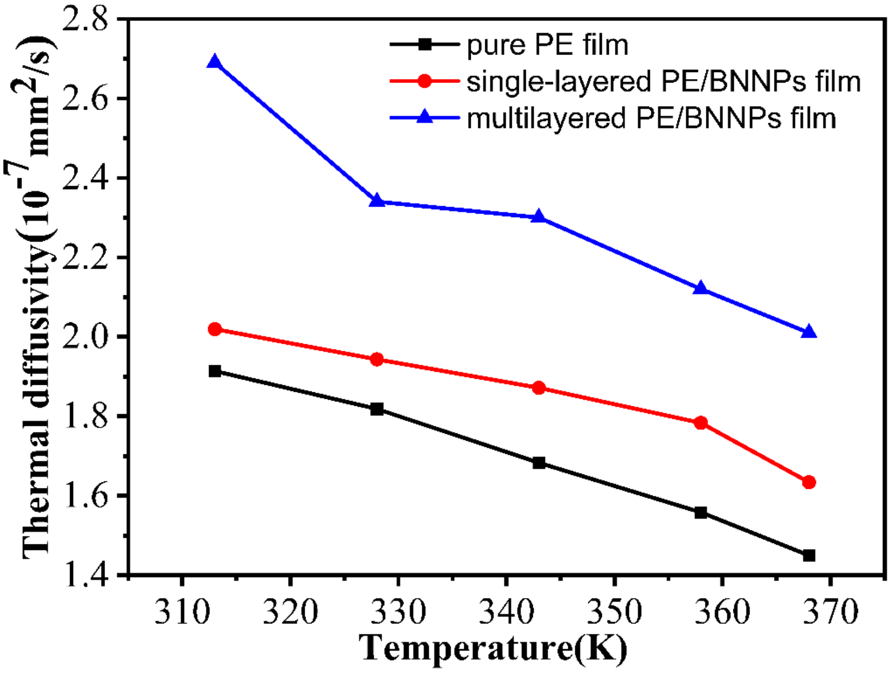

Figure S3. The calculated in-plane thermal diffusivity of different samples.

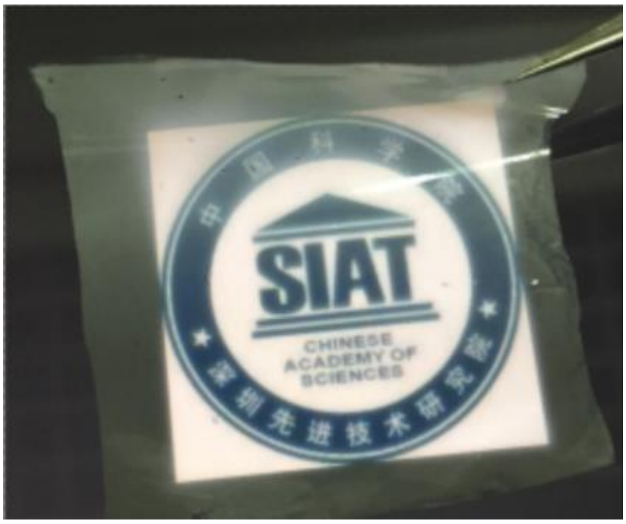

Figure S4. The optical photo of single-layered PE/BNNPs film.

The specific breakdown voltage was calculated from the Weibull distribution, as shown in following formula:

$$P = 1 - exp\left[-\left(\frac{E}{E_0}\right)^{\beta}\right] \quad (1)$$

Deriving this formula, the following formula can be obtained:

$$\ln[-\ln(1-P)] = \beta \ln E - \beta \ln E_0 \quad (2)$$

where P is the cumulative probability density of the electrical breakdown of the sample, β is the shape factor, E_0 is the breakdown voltage. Equation 2 is the basis of the linear fitting in Figures S5–S7, where the slope and intercept can be used to calculate the breakdown voltage E_0. The errors induced from the linear fitting to the slope β and the intercept $b = \beta \ln E_0$ has been considered in the following calculation of the breakdown voltage.

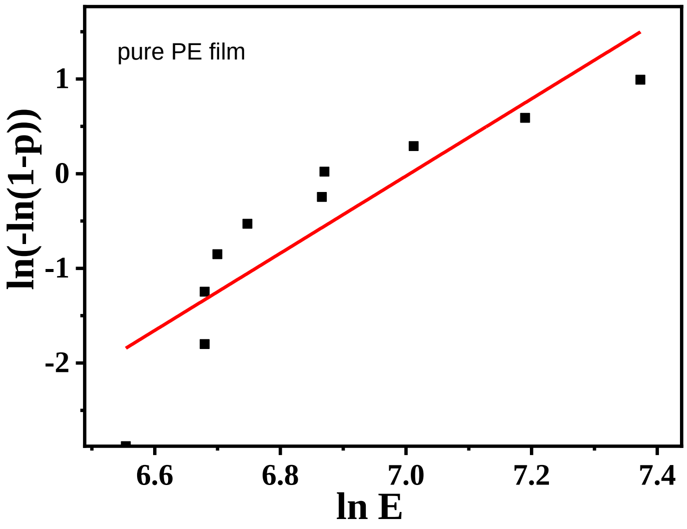

Figure S5. Linear fitting of $\ln[-\ln(1-P)]$ versus $\ln E$ of pure PE film. (The slope is 4.08 ± 0.73, the intercept is −28.58 ±5 .01 and the corresponding R-square value is 77.06%.).

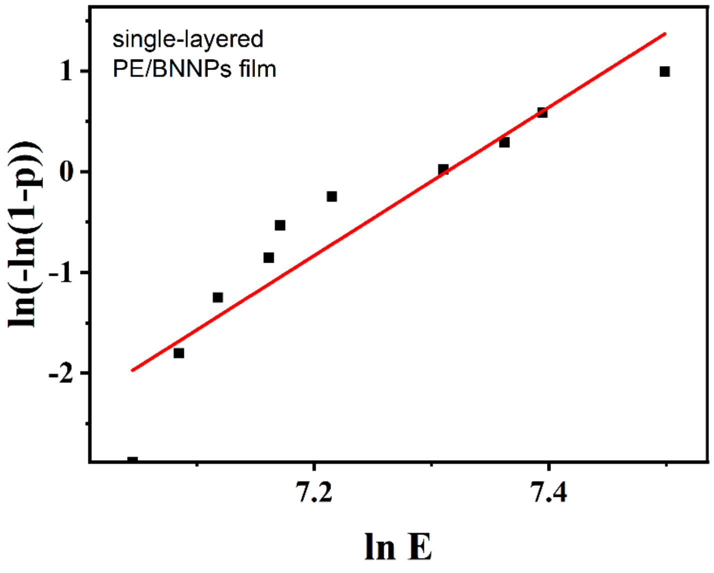

Figure S6. Linear fitting of $\ln[-\ln(1-P)]$ versus $\ln E$ of single-layered PE/BNNPs film. (The slope is 7.36 ± 1.01, the intercept is −53.82 ± 7.21 and the corresponding R-square value is 85.61%.).

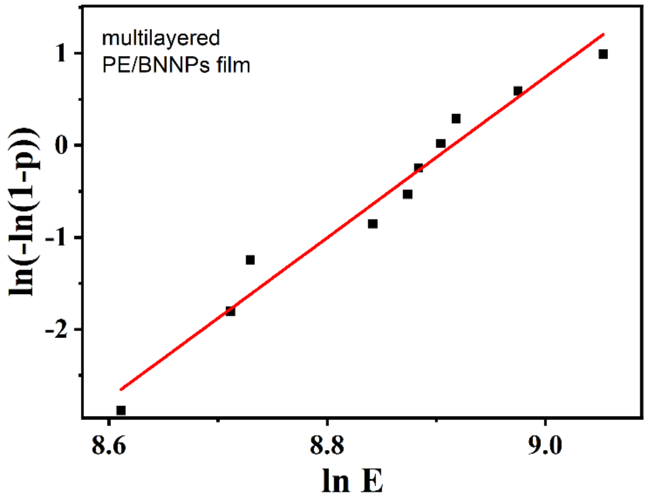

Figure S7. Linear fitting of $\ln[-\ln(1-P)]$ versus $\ln E$ of multilayered PE/BNNPs film. (The slope is 8.73 ± 0.56, the intercept is −77.83 ± 4.98 and the corresponding R-square value is 96.39%.).
